# Inhibitory Control and Fall Prevention: Why Stopping Matters

**DOI:** 10.3389/fneur.2022.853787

**Published:** 2022-03-30

**Authors:** David A. E. Bolton, James K. Richardson

**Affiliations:** ^1^Department of Kinesiology and Health Science, Utah State University, Logan, UT, United States; ^2^Department of Physical Medicine and Rehabilitation, University of Michigan, Ann Arbor, MI, United States

**Keywords:** stopping, response inhibition, aging, balance, falls

## Introduction

The fact that weak leg muscles, impaired vision, numb feet, or insufficient blood pressure can predispose someone to a fall is unsurprising. Consequently, such factors are a standard part of clinical fall risk assessments. However, the relationship between cognitive ability and falls (1–3) is less readily appreciated. And even less intuitive are findings that executive function tests which emphasize inhibitory control, such as a go/no-go or Stroop task, are especially predictive of falls in community dwelling older adults (4–7). This begs the question: “*How does an ability to say “green” when the word “red” is written in green ink on the Stroop evaluation prevent a fall*?” Although not immediately clear, the fog lifts when we consider that inhibitory control is a prerequisite for behavioral flexibility (8). A capacity to inhibit implies that we are no longer at the mercy of a highly automatic response and can instead abort that response allowing adaptation to novel and complex scenarios on short notice. Stopping automatic behavioral tendencies in daily life is critical when such tendencies put us in harm's way and must be quickly modified (e.g., preventing a step that is destined to land on a slippery or unstable surface). Recent insights have shed light on how short latency inhibitory control plays a role in resisting a fall, and in this paper, we advocate for the inclusion of this important concept in clinical assessments and interventions, and intensification of research efforts into its underlying mechanisms.

## The Value of Inhibition in the Control of Upright Posture

Although strong relationships between performance on executive function tests and fall prevalence are suggestive, these in isolation fail to identify the mechanism by which inhibitory control diminishes fall risk. Inhibitory processes range from afferent/perceptual to efferent/motor and commonly decline with age independent of changes in processing speed (9). It is likely that inhibition reduces fall risk through both mechanisms. Perceptual inhibition allows rapid attention to the most salient of multiple stimuli during a postural challenge, while efferent inhibition allows termination of the usual walking motor pattern which becomes obsolete at the instant of perturbation. This may also involve disengaging upper limbs from a secondary task (e.g., holding onto a cane, or groceries) so that the arms can contribute to the more pressing demand for balance recovery (10, 11). Accordingly, there is evidence that efficient afferent inhibition in older adults leads to improved balance performance in situations involving multiple stimuli (12), and that older adults take twice as long to perceive fall events as compared to young people (13). Together these suggest an age-related degradation of the capacity to quickly inhibit background stimuli and thoughts to attend to a falling event quickly.

**Figure 1:**
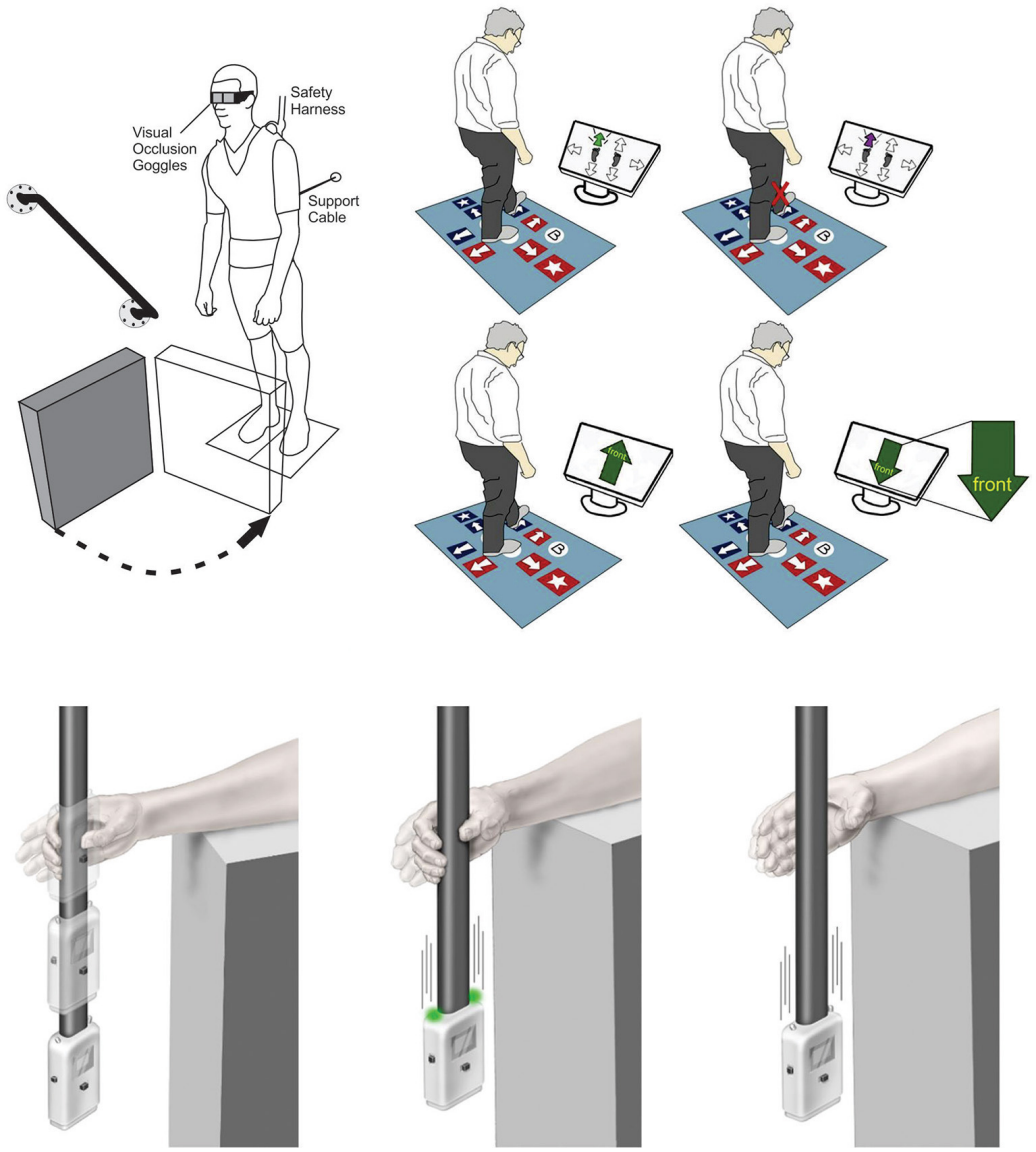
Examples for current methods to assess response inhibition as it relates to balance. Top Left: Release from a supported lean causes a participant to fall forward prompting a compensatory balance reaction. In between attempts, vision is occluded using liquid crystal occlusion spectacles so that objects in the foreground can be rearranged. On some trials a leg block is imposed to force suppression of a highly automatic balance recovery step [Adapted from (14)]. Top Right: Computerized step test used to assess voluntary step reactions to visual cues. Tests include choice stepping reaction time, inhibitory choice stepping reaction time (i.e., suppress step response when purple arrow appears, congruent Stroop step test, and an incongruent Stroop step test (i.e., always step by the word) [Adapted from (5)]. Bottom: ReacStick to assess reaction time. Left Panel: ReacStick Simple Reaction Time (SRT) Test. The device is released, and the participant needs to catch it as quickly as possible. Middle Panel: ReacStick Recognition Reaction Time Test showing the condition where lights illuminate as the device is released which is the indicator for the participant to catch the device. Right Panel: ReacStick Recognition Reaction Time Test showing the condition where lights do not illuminate when the device is released and the participant must resist the urge to catch it [Adapted from (15)]. Importantly, in each experimental paradigm, rapid inhibition of a pre-potent response was required to successfully complete the experimental trial. Accordingly, each of these experimental outcomes predicted successful response to a perturbation and/or reduced fall risk.

Detailed assessment has also exposed efferent inhibitory control deficits in older adults when they perform reactive stepping tasks. Excellent examples were offered by two research teams (16, 17) as they revealed impairments in response inhibition during reactive stepping tasks, particularly when choice was imposed. A strength of these studies was the use of force plates to detect subtle errors in anticipatory postural adjustments preceding a step. This approach offered a sensitive way to measure the postural shift opposite the selected step leg, capturing the step decision latency. Cohen et al. (16) included a Stroop test and showed that Stroop performance correlated with errors in anticipatory postural adjustments. This work suggests that a deficit in response inhibition underlies a choice reaction time delay in older adults. Presumably, such delays would be even more impactful in the time-pressured context of reactive balance control which requires response initiation within 300 ms to avoid hip fracture forces (18). Another group established that performance on a choice stepping task with an inhibitory component was predictive of falls over 1 year, providing a direct link between inhibitory stepping deficits and actual falls (5) (see Figure 1, top right). Key insights emerging from these studies are that response inhibition can be expressed (and assessed) through voluntary step reactions and performance on these tests relates to real-world falls. These tests also offer a plausible means by which deficient inhibition can impair balance recovery and increase fall risk.

The neural control of stopping has been a focus in traditional cognitive neuroscience for decades spurring the development of tests that emphasize suppression of a prepotent response (19, 20). Of these, the stop signal task (SST) is considered a gold standard as it offers a way to quantify the covert stopping process (14, 21). Using this approach, a faster stop signal reaction time (SSRT) indicates a greater capacity to cancel an already initiated response and inhibition as measured by the SST appears to generalize to whole-body postural control (15, 22). Here, participants released from a forward lean posture were challenged to suppress an automatic forward step balance reaction on the minority of trials when a leg block suddenly rendered such a step erroneous (see Figure 1, top left). Importantly, participants with a faster SSRT were better able to suppress the automatic balance recovery step during leg block trials (22). This research bridges performance on an inhibitory cognitive assessment with a balance recovery skill relevant to fall avoidance in a complex real-world setting. Notably, balance recovery in these lean and release studies required more than response inhibition in isolation since participants needed to switch from stepping to an entirely new compensatory arm reaction. Nevertheless, neural imaging has shown that the same neural mechanisms required for rapid stopping also support response switching (23). This is consistent with the idea that inhibition is foundational for cognitive flexibility (8), which is often tested with set-shifting tasks such as the Wisconsin Card Sorting Task (24, 25). In the study of balance control, the concept of switching has also been explored by imposing competing task demands. These include, for example, visuo-motor tracking tasks that require quick reallocation of attention to contend with a sudden postural perturbation (26) or situations where people need to release an object so the hand can grasp a support rail (11). In both cases one must first disengage from a given task before engaging with the next, and delays in switching lead to a less effective corrective response. Thus, the value of inhibition extends beyond simple suppression of an unwanted act to encompass the much broader concept of set-switching and flexible attention allocation. This latter point is of practical interest given the link between mental flexibility and fall prevalence (6).

In recent years, a device called the ReacStick® has been developed with the intent of providing a clinical measure of reaction time and short latency inhibitory executive function (27) (see Figure 1, bottom). This test uses a “ruler-drop” paradigm to measure both simple reaction time and go/no-go reaction accuracy within 390 ms (when the device is dropped from standard desk height). When evaluating reaction accuracy, the participant must either catch, or let the falling device drop, for each trial based on the random illumination of lights affixed to the device, with the outcome being the percentage of 10 light-off trials appropriately not caught. This measure of short latency response inhibition was the strongest predictor of the ability of older adults to recover from laboratory-based trip/slip perturbations (28) and older adults with diabetic neuropathy to maintain frontal plane control when walking on an uneven surface (29). In neither study did lower limb sensorimotor functions influence reactive balance control to the degree of “light off accuracy” suggesting the primacy of inhibitory speed for generating safe, functional recoveries from sagittal and frontal plane perturbations while walking. The finding that short latency inhibitory function predicted falls while simple reaction time did not, highlights the unique value of this mental ability beyond basic processing speed. The simplicity of this device and its potential use as a bedside assessment tool with outcomes that are readily apparent to patient, family, and clinician are important practical considerations, but perhaps most critical is the fact that it forces an urgent response to grasp a rapidly falling object similar to the contracted timeframe available for avoiding a fall or fracture (18). The intense time pressure associated with the falling ReacStick® compels a visceral “Go” response in a manner absent from other cognitive assessments, yet this heightened time pressure is relevant in the domain of falls because it mimics the absolute timing required to respond successfully to an accelerating center of mass before it exceeds a point of no return. Thus, inhibition under temporal constraint would seem an important consideration for accurate fall-risk assessment as well as task-specific training. Ostensibly the ReacStick® may seem less relevant as a fall risk indicator given how it focuses on hand responses rather than the usual trunk and leg responses that come to mind with postural control. Aside from the fact that this test seems to tap into a general capacity for stopping, the upper limbs are frequently used to regain balance particularly in settings where a step reaction is prohibited (30), highlighting the direct utility of this test.

Precisely when neural stopping networks can influence a postural response is not clear. Drawing from recent neuroimaging studies using the SST, early brain markers of response inhibition are evident by ~120 ms after a stop cue, followed by suppression of muscle activity by ~160 ms (31). This provides some indication of the speed at which a prepotent action can be suppressed, however estimates of neural processes based on voluntary reactions would not directly translate to perturbation-evoked reactions which are considerably faster (32). The precise timing of inhibitory processes is further obscured because predictability and prior experience influence top-down control of “lower” reflexes. Here, automatic reflexes can be tuned by executive set and shaped to meet specific contexts. As an example, people learn to suppress a rapid soleus stretch reflex in response to a toes-up tilt perturbation since this reflex is destabilizing in this context (33). Reactive and proactive modes of inhibition are likely both important in effective balance regulation, but detailed mechanisms and precise chronology are presently lacking.

## Discussion

From a basic research standpoint, we need to understand mechanisms of inhibitory control as they pertain to fall prevention. Neural imaging using response inhibition tasks, such as the SST, has revealed specific stopping networks in the brain and captured neural signatures (e.g., Beta bursts) predictive of successful action suppression (31, 34). Given the behavioral evidence hinting that these same neural networks can influence corrective balance reactions (15, 22), traditional cognitive neuroscience offers a useful template for gait and posture research. The challenge here is to translate techniques developed with seated participants into applications which include ballistic, whole-body balance reactions. To determine how inhibitory control impacts postural responses, testing environments must force a need for inhibition, since the key with inhibitory interference control is to override stereotypical but dysfunctional tendencies such as maintaining usual step length following a trip or releasing a handheld object to allow a compensatory grasp onto a support rail (10, 11). That means, in addition to leveraging neural imaging approaches from cognitive neuroscience, there is a need to incorporate experimental designs where reflexive or typical action must be suppressed. Hannah and Aron (35) recently discussed the methodological hurdles to overcome when assessing stopping ability in real-world scenarios along with the value of this research. Developing such methods could help pinpoint specific deficiencies in the neural control of balance, especially in situations that demand behavioral flexibility to avoid a fall. Although in the present article we have focused on the ability of inhibitory brain processes to suppress an inappropriate motor response to a balance challenge, it's also likely that these same neurocognitive resources could preemptively help us avoid precarious situations in the first place (36).

At the clinical level, evaluations that emphasize inhibition would provide valuable insight into an established marker for fall risk. Measuring this ability to stop and change a routine movement pattern is particularly relevant in light of the recent meta-analysis by Nørgaard et al. (37) showing that Gait Adaptability Training was superior to standard activity programs (e.g., strength and balance training) in reducing falls in community-dwelling older adults. These findings highlight the value of incorporating an inhibition demand into reactive balance training to tax and train these skills. This is consistent with recent insights from a scoping review where the authors showed the significance of inhibitory control when it's required for successful balance performance, and this was true despite a lack of standardization for tasks and outcome measures used in the few studies on this topic to date (38). An excellent example for how this type of training could be accomplished is via the Computer Assisted Rehabilitation Environment, or CAREN® system, which uses virtual reality technology to introduce real world scenarios and combines this with multi-axis perturbations during treadmill walking (39). Indeed, findings that neural networks serving balance can be engaged through mental imagery (40–42) suggest that executive processes such as response inhibition could even be trained to improve fall resistance without physical risk. While complicated lab settings and costly equipment may not be feasible on a large scale, it may still be manageable to use tests like a voluntary choice reactive step task (5), the ReacStick® (27) or computerized executive function tests (e.g., go/no-go, or SST) to complement a clinician's toolkit. Beyond outright inclusion of a test for inhibition, *timing* of such screening should also be considered. This idea is consistent with suggestions that screening for potential risk factors should occur in midlife before falling becomes a pressing issue (43), and particularly after the initiation of medications with anticholinergic potency due to their impact on executive function (44). Given that inhibitory executive functions decline by middle age (45), this may represent an early marker for fall risk analogous to elevated blood pressure being a treatable risk factor in advance of cardiovascular events. It is interesting to note that in a 5-year prospective study, lower performance on executive function tests that emphasized inhibition was predictive of future falls, even in cognitively intact older adults (4). Thus, inhibition may serve as a canary in the mine shaft when it comes to gauging fall risk in future years.

Roughly a decade ago, Lui-Ambrose and colleagues argued that reduced falls from traditional training approaches such as the Otago Exercise Program may be at least partly mediated through improved executive functions, including inhibitory control (46). This was an important insight as the authors looked beyond the widespread assumption that physical strength was primary. While sufficient strength is undoubtedly useful to prevent a fall, convergence from multiple lines of evidence now shows inhibitory control as a significant and unique factor in fall prevention. This finding will impact clinical practice as caregivers seek to identify inhibitory deficits and when found, critically appraise medications for anticholinergic potency, consider sleep evaluations, or possibly recommend stimulants. This approach to fall risk assessment identifies specific risk factors allowing for targeted remediation, rather than generic referrals for physiotherapy. In terms of basic research, a major goal would be to map out neural activity similar to what has been accomplished in cognitive neuroscience but using experimental designs relevant to prevent a fall. Overall, this opinion article presents a view on the increasingly evident value of rapid inhibition in controlling balance and proposes this as a useful concept to include in assessment and treatment to reduce fall risk. This message comes at a time where the limits of current practice in reducing falls in an aging society are becoming apparent (47).

## Author Contributions

Both authors listed have made a substantial, direct, and intellectual contribution to the work and approved it for publication.

## Funding

The Newman Family Foundation supports JR's academic pursuits.

## Conflict of Interest

JR is a co-inventor of ReacStick. Additionally, his son has created an LLC (Mineurva) which is currently marketing ReacStick to researchers and seeking FDA clearance for the device. However, JR does not have a direct financial interest in Mineurva. The remaining author declares that the research was conducted in the absence of any commercial or financial relationships that could be construed as a potential conflict of interest.

## Publisher's Note

All claims expressed in this article are solely those of the authors and do not necessarily represent those of their affiliated organizations, or those of the publisher, the editors and the reviewers. Any product that may be evaluated in this article, or claim that may be made by its manufacturer, is not guaranteed or endorsed by the publisher.
